# Editorial: Ageing-Related Symptoms, Kampo Medicine, and Treatment

**DOI:** 10.3389/fnut.2021.749320

**Published:** 2021-11-01

**Authors:** Masahiro Ohsawa, Yasuhito Uezono, Akio Inui

**Affiliations:** ^1^Department of Neuropharmacology, Graduate School of Pharmaceutical Sciences, Nagoya City University, Nagoya, Japan; ^2^Department of Pain Control Research, Jikei University School of Medicine, Tokyo, Japan; ^3^Pharmacological Department of Herbal Medicine, Kagoshima University Graduate School of Medical & Dental Sciences, Kagoshima, Japan

**Keywords:** herbal medicine, Japanese Kampo medicine, aging-related disease, longevity, frail

## An Aging Population Is a Critical Problem Facing the Entire World

The number of individuals aged 60 years and older in the population is increasing worldwide, and was reported by the World Health Organization to be one billion worldwide in 2019 (1). This number will increase to 1.4 billion by 2030 and 2.1 billion by 2050, meaning that the aging of the population will progress rapidly over the next half a century. According to the estimated rate of aging in each region, the aging population is expected to increase rapidly, not only in developed countries, but also in developing countries.

Concurrent with an aging population, birth rate has also been declining significantly. To maintain economic activities in the face of a declining future population, ensuring the health and activities of older adults will become increasingly important. In particular, the aging of the population accompanied by a low birth rate is expected to continue in developed countries, such as the United States, China, and Europe, as well as in some developing countries. Facilitating an age-friendly world requires an essential and urgent response to our changing demographics, for example, by including health and social care, transportation, housing, and urban planning.

## The Importance of Kampo Medicine For Resolving Age-Related Symptoms

Kampo medicine utilizes herbal formulas derived from classical Chinese medicine that originated ~3000 years ago and developed in Japan (2). Classical Chinese medicine was brought to Japan in the fifth and sixth centuries and was modified to fit the Japanese climate and the characteristics of Japanese people. Different formulations of Kampo medicine are prescribed based on the International Classification of Diseases-10 diagnosis, similar to Western medicine that uses prescribed drugs for symptoms. Importantly, Kampo medicine formulations are composed of multiple herbs, and the ratio of each herb is calculated based on experience accumulated over 1000 years.

The beneficial effects of Kampo medicine have been identified in basic and clinical research. For example, as published in our previous research topic, Kampo medicine is effective in treating anorexia, loss of skeletal muscle mass, and decreased activity in older adults (3). This research topic follows on from our previous one titled “Frailty and herbal medicine—-from molecular mechanisms to clinical efficacy” (3). In this Research Topic, Takayama et al. summarized the recent advances in Kampo medicine for the management of symptoms related to functional decline of the cardiovascular, respiratory, and digestive systems, cognitive impairment and related disorders, pain, and other sensory issues, among others.

## The Quality of Kampo Medicine

The main problem using Kampo medicine is the misunderstanding relating to its validity. Differences in the validity of different Kampo medicine formulations result from the manufacturing variations because there is no gold standard as a reference to control the quality of Kampo medicinal formulations. In Japan, the amounts of active components in Kampo medicine are strictly regulated. Wang et al. characterized the ingredients of various commercially available traditional crude drugs, *shinkiku*, which are prepared by microbial fermentation of wheat and herbs. They showed that digestive enzymes, organic acids, and 39 volatile compounds were commonly found in the products. However, the chemical and microbial characteristics differed according to the manufacture process. Interestingly, the dominant ingredient in Korean products differs from that in Chinese products. There are both commonalities and diversities among the commercially available *shinkiku*. The commonalities may serve as reference standards for quality control of *shinkiku*. In contrast, the diversities suggest the importance of microbial management in stabilizing the quality of *shinkiku*.

## Beneficial Effects of Kampo Medicine For Age-Related Symptoms

Kampo medicine has been shown to improve various bodily functions. For geriatric syndromes, ninjin'yoeito (NYT) and yokukansan-ka-chimpihange (YKSCH) are often prescribed to improve symptoms such as fatigue, anemia, anorexia, night sweats, cold limbs, slight fever, chills, persistent cough, malaise, mental disequilibrium, and insomnia. NYT activates the hypothalamic neuropeptide Y (NPY) neurons through the activation of voltage-gated Ca^2+^ channels (Goswami et al.). Interestingly, NYT preferentially affects the N-type and L-type Ca^2+^ channels in ghrelin-sensitive and ghrelin-insensitive neurons, respectively. Moreover, NYT activates the orexin 1 receptors (Miyano et al.). These two important basic studies implicate the orexigenic effects of NYT. NYT also alleviates neuropathic pain (Takemoto et al.) and suppresses the progression of age-related hearing loss (Kawashima et al.), suggesting that NYT could improve the undesirable symptoms of aging. In addition to NYT, YKSCH improves sleep disruption and reduces the levels of allopregnanolone, a positive allosteric modulator of GABA_A_ receptor (Murata et al.). Taken together, these results strongly suggest that Kampo medicine has the potential to improve geriatric syndrome.

## Clinical Studies Support the Effectiveness of Kampo Medicine For Age-Related Symptoms

The clinical case reports in this research topic demonstrate many beneficial effects of Kampo medicine, especially NYT and YKSCH. NYT has been shown to improve the quality of life of frail patients after hospitalization (Kashima). NYT also increased body weight and muscle mass in patients with dementia (Matsui and Matsui). Furthermore, NYT has demonstrated great outcomes in terms of muscle mass, appetite, and body weight (Morinaga et al.). Notably, rikkunshito was ineffective in NYT-sensitive patients with hip fracture and sarcopenia. These results suggest that the appropriate choice of Kampo medicine formulation based on the patient's condition is important for improving geriatric symptoms. NYT also improved the subjective symptoms and nutritional status of patients with idiopathic pulmonary fibrosis (Kushima et al.). In the case of adjuvant chemotherapy regimen, NYT reduced the adverse effects of anticancer therapy, particularly in older patients (Aomatsu et al.) Therefore, NYT can improve several geriatric syndromes in several disease conditions.

In addition to NYT, YKSHC also improved REM sleep behavior disorder in Lewy body dementia (Manabe), aggressive disorder, and sleep disorder in patients with dementia (Katsumoto et al.). Hachimijiogan (HJG) also improved irregular menstruation in young to middle-aged women (Hirabayashi). This effect of HJG is more prominent in crude drug preparations than in HJG extract preparations.

## Further Considerations of the Effectiveness of Kampo Medicine For Age-Related Symptoms

This research topic contains many clinical case reports supporting the beneficial effects of several different Kampo herbs and preparations across patients of different ages. The key limitations of these research studies include the small sample sizes and the lack of a double-blind randomized controlled trial (RCT). A double-blind RCT for NYT is currently underway in Japan. In the future, we hope that a large RCT will be conducted based on the findings reported in this research topic. Our previously published research topic may also be informative for readers interested in herbal medicines (3). The past and present research topics can provide further insights into the use of Kampo medicine for improving the quality of life in older adults.

## Author Contributions

MO wrote the manuscript. AI conceived and organized the structure of the editorial. MO, AI, and YU contributed to the critical reading of the first draft version, revision, and approved the final manuscript for publication. All authors contributed to the article and approved the submitted version.

## Conflict of Interest

The authors declare that the research was conducted in the absence of any commercial or financial relationships that could be construed as a potential conflict of interest.

## Publisher's Note

All claims expressed in this article are solely those of the authors and do not necessarily represent those of their affiliated organizations, or those of the publisher, the editors and the reviewers. Any product that may be evaluated in this article, or claim that may be made by its manufacturer, is not guaranteed or endorsed by the publisher.
